# Negative Predictors of Outcomes of Flexor Tendon Repairs

**DOI:** 10.7759/cureus.4303

**Published:** 2019-03-23

**Authors:** C M Hurley, Frank Reilly, Simon Callaghan, MN Baig

**Affiliations:** 1 Plastic Surgery, University Hospital Galway, Galway, IRL; 2 Orthopaedics, University Hospital Galway, Galway, IRL

**Keywords:** flexor tendon repair, negative predictor, timing, smoking, age

## Abstract

The current trend in hand surgery has streamlined the treatment of acute hand trauma to the modern-day surgery unit. As the volume of hand trauma caseloads continues to increase, it is becoming increasingly difficult to schedule patients for theater on the day of injury. It, therefore, becomes paramount to adequately triage patients in accordance with best clinical evidence and predictors of poor clinical outcomes.

Animal models suggest that the earlier flexor tendons are repaired, the better the patient functional outcome. The largest study to date examining the timing of injury to functional post-operative outcome also recognizes that the faster these injuries are repaired, the better the patient outcome. Age-related changes to tendon biomechanics and structure are well-documented. However, no conclusive evidence exists specific to the degenerative changes and mechanical properties of flexor tendons in humans. The animal model strongly suggests that increasing age is associated with local architectural and biological changes that directly affect the tendon repair functional outcome. Although retrospective analyses to date suggest that smoking is a negative outcome predictor for functional tendon outcome, no prospective large-scale studies exist.

A large, single-center prospective study specifically examining the positive and negative outcome predictors of flexor tendon repairs and functional post-operative outcome is warranted. The negative predictive model of patient care may enable us to further council patients preoperatively and stratify patients according to clinical need.

## Introduction and background

The core of hand surgery research is focused on improved suture techniques and postoperative rehabilitation protocols [1]. Despite an overwhelming amount of investigation over recent years in the subject matter, changes in surgical practice are stagnant. The objective of appropriate flexor tendon repair includes adequate strength, nominal gapping, and comfortable tendon gliding and excursion [1-2]. Flexor tendon injuries are one of the most common hand injuries encountered in the plastic surgery day unit and continue to increase in incidence [3]. Day-case surgery is an effectual modality of treating hand trauma [4]. However, with this model, patients are triaged, given outpatient appointments, and may be waiting longer for surgery than the traditional model of care. Only 52% of plastic surgery hand trauma units in the United Kingdom have a dedicated weekend theater list, leading to patients with significant injuries potentially waiting days for surgical repair [5]. It, therefore, becomes paramount to adequately triage patients in accordance with best clinical evidence and predictors of poor clinical outcomes.

Predictors of poor outcomes after flexor tendon repairs have long been debated [6-8]. Age, smoking, injury location, soft tissue damage, local vascular injury, and skeletal injury remain objective parameters and indicators of patient outcome. The purpose of this paper is to evaluate the negative predictors of functional outcome in flexor tendon repairs, delay of surgery, age, smoking status, and zone of injury.

## Review

Timing of surgery

The effect of the timing of repair and the functional outcome of flexor tendons remains controversial and underreported. The solitary animal model data of timing of surgery in flexor tendon repairs is decades old. Tang et al. report a chicken model of flexor tendons repaired at intervals of one, four, eight, 14, and 20 days after injury (Table 1) [9]. In this model, the flexor sheath was closed primarily on one foot and surgically excised on the other. The authors report that tendon excursion, motion, and morphology are best when repaired primarily, and decline in success the longer the delay in surgery. Interestingly, sheath closure was associated with poorer clinical outcome, especially in the delayed cohort. Similar results were demonstrated by Gorriz et al. in a series of chicken flexor tendons [10]. The best functional outcomes were tendon repairs immediately after injury. The next-best repairs were after 10 days with the worst clinical outcomes between four and seven days.

**Table 1:** Research studies describing the timing of flexor tendon repairs N/A = Not Applicable

Reference	Year	Subjects	No. of tendons	Risk factor
Gorriz et al. [10]	1976	Chickens	30	Timing of surgery
Tang et al. [9]	1995	Chickens	60	Timing of surgery
Ackerman et al. [19]	2017	Mice	N/A	Age
Tottenham et al. [11]	1995	Humans	22	Timing of surgery
Kasashima et al. [7]	2002	Humans	29	Age, timing, zone, vascular injury, rehabilitation
Rigo et al. [6]	2016	Humans	322	Age, timing, smoking, zone, vascular injury

Range of motion is one of the most frequently reported measures used in clinical practice to report the outcomes of flexor tendon repairs [2]. The importance of the timing of surgical intervention on active range of motion was first investigated by Tottenham et al. (Table 1) [11]. All 22 patients included followed a standard postoperative regime. Interestingly, patients in the early intervention group had a better total active moment (TAM) of both the proximal interphalangeal joint (PIPJ) and distal interphalangeal joints (DIPJ) than delayed repairs, but this was not statistically significant. Strickland's classification was used by Tottenham et al. to grade the percentage of the normal range of motion [12]. All patients in the early surgical repair cohort exhibited “Excellent” or “Good” on the Likert scale, whereas three patients in the delayed group demonstrated “Fair” or “Poor” results.

In contrast, Rigo et al.’s retrospective examination of 356 flexor tendon repairs in 291 patients defines delay to surgery as a surgical intervention at 14 days or more post-injury [6]. They assessed patients eight weeks postoperatively and at their final clinic visit (mean=seven months). Strickland's classification was used to document TAM [12]. Although delay of surgery was unable to show any direct patient effect, including it into the regression equation improved the statistical model.

Kasashima et al. examine 29 flexor pollicis longus tendons at variable degrees of delay to surgical intervention. Ten tendons were operated immediately, six within a week, eight within three weeks, and five delayed more than a week. The mean follow-up was 3.1 years (mean = six months to 12 years). Eight tendons were zone I, 14 were zone II, and seven were zone III repairs. Functional evaluation of tendons was assessed using a Likert scale. As with Rigo et al.’s cohort, there was no statistical significance in TAM and delay of surgery. Half of the immediate tendon repairs received “Excellent” or “Good” outcomes, with half having “Fair” or “Poor” outcomes. Again, this study suffers from a small patient cohort, with a smaller number of candidates in the delayed tendon repair category. Secondary to the nature of retrospective study design, its significant difference of follow-up timescale greatly distorts the TAM assessment. As the tendon repairs were not limited to one zone of injury, it is difficult to compare any results with accuracy. In zone II of the thumb, there is a pulley system and a local avascular region of the tendon [13] and is typically known as “no man’s land.” These are typically associated with a poorer clinical outcome, including TAM [14].

Age

The effects of aging on the biomechanical properties during homeostasis, healing potential, and repair rupture rate have been well-documented in the rotator cuff tendon [15-16], patellar tendon [17], and Achilles tendon [18]. It has been demonstrated in animal models that flexor tendon healing is impaired with increasing age [19]. With an increase in age, tendons are predisposed to a decrease in matrix deposition, resulting in a decline of their mechanical properties. Ackerman et al. examine the mechanical properties of flexor digitorum longus, flexor carpi ulnaris, and tail fascicles in both male and female C57B1/6 mice between three and 27 months (Table 1). Interestingly, no change in max load at failure was observed in any age group of mice. However, they identified a significant loss of bridging collagen extracellular matrix in tendon repairs of aged mice. This suggests that matrix production may lead to impaired tendon healing with increasing age.

There is a paucity of data strictly examining age as a predictor for flexor tendon repair outcomes in the human model. Kasashima et al.’s examination of 29 flexor pollicis longus tendons stratified the cohort into 10-20 years, 21-30 years, 31-40 years, and more than 41 years. The authors hypothesized an age of 20 or less as a potential safe factor and an age of over 20 as a potential risk factor. However, for any combination of clinical state, including the timing of surgery, zone of injury, vascular injury, and postoperative management, age was not a predictor of outcome.

Contrary to Kasashima et al.’s findings, Rigo et al. report that increasing age may be a significant negative predictor at eight weeks for the postoperative active range of flexor tendon repairs [6]. This is across all zones of injury. Interestingly, his multivariate regression analysis predicts a final decrease of 0.7 degrees of active range of motion with every additional year of age. These findings are in keeping with previous animal models [19], and this is the largest study to date examining age as a possible predictor of flexor tendon outcome.

Smoking

Smoking has been well-documented to have negative clinical effects on the musculoskeletal system, including increased rates of tendon rupture, soft tissue infection, wound-healing complications, and a negative influence on clinical outcomes [20]. Smoking is implicated in a number of negative systemic effects on soft tissue healing; reduced blood supply, tissue hypoxia, and the effects of nicotine on arteriole endothelial receptors have been demonstrated to directly decrease tendon metabolic activity in smokers [21-22]. It is well-reported that fibroblasts, mesenchymal stem cells, acute phase proteins, and growth factors are needed to form granulation tissue [21]. A single cigarette has been implicated in a digital artery blood velocity and volumetric flow decrease, increasing vascular resistance and overall tissue perfusion [23]. Although the effects of smoking on this routine wound healing model is directly implicated in poorer fracture healing outcomes and bone fusion processes, it’s action on tendon healing is less studied.

To the author's knowledge, there have been no animal model studies on the effects of nicotine specific to flexor tendon healing. Galatz et al. examine the effects of nicotine on rotator cuff injury and repair with the use of the rodent model [24]. In this study of 72 rats, inflammation persisted longer in the nicotine cohort. Cellular proliferation was lower in the nicotine group, with a lower expression of type-1 collagen. Overall, Galatz et al. report that nicotine caused a delay in tendon healing. This important clinical outcome is clinically correlated in Rigo et al.’s report of smoking as a negative outcome predictor in his model of 322 flexor tendons. Positive smoking status was represented by a decrease in active range of motion by up to 29 degrees overall at eight weeks postoperatively. Smoking status was on par with an associated phalangeal fracture as a predictor of a negative outcome for the patient.

Zone of injury

Zone II injuries are well-established risk factors for poorer functional outcomes [6-8]. Zone II injuries have been associated as the most complicated zone of injury for decades and, therefore, most clinical research is directed at this zone’s surgical approach and clinical outcome [8]. Very few studies have examined the surgical approach to zone 4 or zone 5 and the associated structures that are likely to be damaged with these injuries [25].

The functional outcome of zone 2 injuries has been well-documented. Rigo et al. report that failure to preserve the tendon sheath or pulley was a direct negative predictor of the postoperative range of motion, with a loss of up to 15 degrees at eight weeks. These poorer outcomes are directly related to the zone’s difficult anatomical presentation; both the flexor digitorum profundus and flexor digitorum superficialis run within its fibro-osseous digital sheath [1]. Furthermore, on subgroup analysis, Rigo et al. report subzone 2C as the most demanding location, with injuries between the A2 and A4 pulleys presenting the worst functional outcome. Kasashima et al. summarized similar data, with tendon repairs in zone II up to 10 times as much as those in zone 1 and zone 3.

Current trends

There are many biological, technical, and surgical problems and challenges with flexor tendon repairs. These challenges that the surgeon's face have yielded little change to the paradigm of clinical research on flexor tendon repair and clinical outcomes in the past decade. Developments in improving global rupture rates, tenolysis rates, and complication rates remain static [8]. Postoperative adherence and tethering, producing a poor functional outcome, subjugate the surgeon’s efforts. Contemporary research in flexor tendon protocol is dominated by the field of rehabilitation. Despite the abundance of data on surgical approach, suturing methods, and rehabilitation programs, a universal best practice does not exist and is largely based on surgical preference [1]. However, our data suggest that patient selection may guide preoperative planning, counseling, and outcome expectations for patients.

The current trend in hand surgery has streamlined the treatment of acute hand trauma to the modern-day surgery unit. As the volume of hand trauma caseload continues to increase, it is becoming increasingly difficult to schedule patients for theater on the day of injury [3]. The British Society for Surgery of the Hand guidelines state that clean flexor tendon divisions may be appropriately repaired up to seven days post-injury. However, animal models suggest that the earlier flexor tendons are repaired, the better the patient's functional outcome. The largest study to date examining the timing of injury and functional postoperative outcome also recognizes that the faster these injuries are repaired, the better the patient outcome [6]. However, Rigo et al.’s work suffers from its retrospective study design and a wide-ranging follow-up period. Although Kasashima et al. found no statistical significance with the inclusion of delay to surgery in the statistical model, Tottenham et al. advocate that early timing of surgery is, at the minimum, suggestive of a positive predictive value for better functional outcome, but larger numbers are needed.

Age-related changes to tendon biomechanics and structure are well-documented. However, no conclusive evidence exists specific to the degenerative changes and mechanical properties of flexor tendons in humans. The animal model strongly suggests that increasing age is associated with local architectural and biological changes that directly affect tendon repair functional outcome [19]. Conflicting data exist in human studies; age-related effects on functional tendon repair outcome are secondary outcomes in retrospective studies that suffer from small numbers [6-7]. Similarly, this restriction applies to our knowledge of smoking status and tendon repair outcomes. Although retrospective analyses to date suggest that smoking is a negative outcome predictor for functional tendon outcome, no prospective, large-scale studies exist. Existing data suggest that preoperative counseling of patients, with an emphasis on smoking cessation, is vital to future functional use of the hand.

## Conclusions

In the current modern trend toward the day surgery trauma unit, patient prioritization is paramount. The negative predictive model of patient care may enable us to further council patients preoperatively and stratify patients according to clinical need. The scheduling of surgeries may be more appropriately stratified according to strong negative predictors of outcome, including age, smoking status, and zone of injury. A large, single-center prospective study specifically examining the positive and negative outcome predictors of flexor tendon repairs and functional postoperative outcome is warranted.
